# Peritoneal macrophages have an impaired immune response in obesity which can be reversed by subsequent weight loss

**DOI:** 10.1136/bmjdrc-2019-000751

**Published:** 2019-11-02

**Authors:** Lisa Willemsen, Annette E Neele, Saskia van der Velden, Koen H M Prange, Myrthe den Toom, Cindy P A A van Roomen, Myrthe E Reiche, Guillermo R Griffith, Marion J J Gijbels, Esther Lutgens, Menno P J de Winther

**Affiliations:** 1 Experimental Vascular Biology, Department of Medical Biochemistry, Amsterdam Cardiovascular Sciences (ACS), Amsterdam UMC-Location AMC, University of Amsterdam, Amsterdam, Netherlands; 2 Departments of Pathology and Molecular Genetics, CARIM School for Cardiovascular Diseases and GROW School for Oncology and Developmental Biology, Maastricht University, Maastricht, Netherlands; 3 Institute for Cardiovascular Prevention (IPEK), Ludwig Maximilians University Munich, Munich, Germany

**Keywords:** type 2 diabetes, obesity, Macrophages, infection control, Weight loss, diet, inflammation

## Abstract

**Introduction:**

Obesity is recognized as a risk factor for various microbial infections. The immune system, which is affected by obesity, plays an important role in the pathophysiology of these infections and other obesity-related comorbidities. Weight loss is considered the most obvious treatment for obesity. However, multiple studies suggest that the comorbidities of obesity may persist after weight loss. Deregulation of immune cells including adipose tissue macrophages of obese individuals has been extensively studied, but how obesity and subsequent weight loss affect immune cell function outside adipose tissue is not well defined.

**Research design and methods:**

Here we investigated the phenotype of non-adipose tissue macrophages by transcriptional characterization of thioglycollate-elicited peritoneal macrophages (PM) from mice with diet-induced obesity and type 2 diabetes (T2D). Subsequently, we defined the characteristics of PMs after weight loss and mimicked a bacterial infection by exposing PMs to lipopolysaccharide.

**Results and conclusions:**

In contrast to the proinflammatory phenotype of adipose tissue macrophages in obesity and T2D, we found a deactivated state of PMs in obesity and T2D. Weight loss could reverse this deactivated macrophage phenotype. Anti-inflammatory characteristics of these non-adipose macrophages may explain why patients with obesity and T2D have an impaired immune response against pathogens. Our data also suggest that losing weight restores macrophage function and thus contributes to the reduction of immune-related comorbidities in patients.

**Significance of this study**

**What is already known about this subject?**

- Obesity is recognized as a risk factor for microbial infections.
- Weight loss is considered the most obvious treatment for obesity.

**What are the new findings?**

- We found a deactivated state of non-adipose tissue macrophages in a mouse model for obesity and type 2 diabetes (T2D).
- Weight loss could reverse this deactivated macrophage phenotype.

**How might these results change the focus of research or clinical practice?**

- The deactivated characteristics of these macrophages may explain why patients with obesity and T2D have an impaired immune response against pathogens.
- Our data suggest weight loss restores macrophage function and thus may lead to a reduction of immune-related comorbidities in patients.

## Introduction

Obesity is characterized by a broad spectrum of obesity-related comorbidities. The most frequently occurring comorbidities are type 2 diabetes (T2D), cancer, cardiovascular diseases, and respiratory and immune dysfunction.^1^ Moreover, obesity has been linked to worse clinical outcomes of bacterial infections like pneumococcal diseases,^2 3^ viral infections such as dengue fever,^4^ and parasitic diseases like malaria.^5^ Furthermore, the duration of influenza A virus shedding in obese adults is increased compared with non-obese adults.^6^ These data indicate disturbed immune activation in obesity and a suppressed ability to fight pathogens.

Previous studies have shown that a modest weight reduction of 5%–10% in patients with obesity-associated complications already leads to health benefits.^7^ However, others indicate that obesity-induced changes, as an elevation in lipid mediators and a depletion of bone marrow-derived mesenchymal progenitor cell subpopulations, persist after weight loss.^8 9^ Therefore, additional treatment may be warranted to reverse all comorbidities.

Immune cell dysfunction in obese adipose tissue is well characterized. In brief, expanded adipose tissue in obesity recruits immune cells that contribute to a chronic state of low-grade inflammation and dysregulated metabolism.^10 11^ Macrophages are key mediators of inflammation and insulin resistance in obesity.^12 13^ In adipose tissue of obese individuals, macrophage populations switch from anti-inflammatory, homeostasis maintaining cells towards a more proinflammatory phenotype with insulin desensitizing actions. However, little is known about macrophage function in non-adipose tissue in obesity, consequences of weight loss and the relation to host defense.

To define the dysregulation of non-adipose tissue macrophages in obesity and after weight loss, we analyzed thioglycollate-elicited peritoneal macrophages (PM) from mice with diet-induced obesity (DIO) and T2D. We performed systemic metabolic and immunological characterization of these mice and linked this to activation and transcriptional profiles of PMs, either under unstimulated conditions or on activation by a pathogen-associated ligand, lipopolysaccharide (LPS).

## Research design and methods

### Mice

Six-week-old male C57BL/6J mice were purchased from Janvier Labs. Mice were housed at the Animal Research Institute Amsterdam UMC (ARIA). Mice were randomly assigned to the experimental groups in disposable IVC Rodent Caging Systems (Innovive) in groups of six mice per cage. Mice were fed a low-fat diet (LFD; 10% kcal fat, D12450B (I), Research Diets) or a high-fat diet (HFD; 60% kcal fat; D12492 (I), Research Diets) ad libitum for 10 or 20 weeks.

### Glucose and insulin tolerance tests

Glucose (GTT) and insulin tolerance tests (ITT) were performed after 8–9 weeks and 18–19 weeks of the diet. Mice fasted for 5 hours for the GTT, and glucose (1 mg/g bodyweight, Sigma-Aldrich) was injected intraperitoneally. For the ITT, mice fasted for 4 hours and insulin (0.75 mU/g bodyweight, Sigma-Aldrich) was injected intraperitoneally. Blood glucose levels were measured using a Bayer Contour glucometer.

### Triglycerides and cholesterol measurements

After 9 and 19 weeks of the diet, mice fasted for 4 hours before blood was collected via the tail vein. Total plasma cholesterol and triglyceride levels were quantified by enzymatic CHOD-PAP and GPO-PAP methods (Roche).

### Histology

Visceral fat and liver were collected, fixed in 4% paraformaldehyde (Sigma-Aldrich) and embedded in paraffin. Four-micron sections were made and stained with H&E. The amount of ‘infiltrating’ immune cells was determined by blinded examination of the sections together with an experienced pathologist and used as a measure of inflammation. Sections were assigned to a ‘Low’, ‘Intermediate’ or ‘High’-grade inflammation group.

### Peritoneal macrophages

After 10 or 20 weeks of the diet, six mice per group received intraperitoneal injections with 3% thioglycollate (Fisher Scientific) to induce a sterile inflammation that attracts monocytes. Mice were euthanized by CO_2_ asphyxiation after 4 days. The peritoneum was flushed with ice-cold phosphate buffered saline (PBS) and cells were collected. Peritoneal cells were cultured in RPMI-1640 containing 25 mM HEPES, 2 mM L-glutamine, 10% fetal calf serum, 100 U/mL penicillin and 100 µg/mL streptomycin (GIBCO). After 3 hours, floating cells were washed away and adherent cells (~95% CD11b^+^ F4/80^+^ macrophages, online supplementary figure S1) were stimulated with or without 100 ng/mL LPS (Sigma) for 3 or 24 hours or 20 ng/mL interleukin-4 (IL-4; Peprotech) for 24 hours. Thioglycollate-injected mice were only used for PMs and not for other analysis.

10.1136/bmjdrc-2019-000751.supp1 Supplementary data

### Cytokine and nitric oxide measurement

After sacrifice, whole blood was collected via the retro-orbital vein in 0.5 M EDTA (Invitrogen) containing tubes. Plasma and supernatant cytokines were detected using an electrochemiluminescence assay. A V-PLEX Proinflammatory Panel 1 mouse kit (MSD) was used to measure tumor necrosis factor (TNF), interferon gamma (IFNγ), C-X-C motif chemokine ligand 1 (CXCL1) and IL-10. Data were acquired using a MESO QuickPlex SQ 120 plate reader (MSD). Nitric oxide (NO) production was assessed by a Griess reaction (Sigma-Aldrich) according to the supplier’s protocol.

### Flow cytometry

To measure surface marker expression, 1.5×10^5^ PMs were seeded per well in a 96-well plate and stimulated with 100 ng/mL LPS or 20 ng/mL IL-4 for 24 hours. After stimulation, PMs were detached with citrate buffer (17 mM tri-Sodium citrate dihydrate and 135 mM potassium chloride in H_2_O). Fc receptors of PMs were blocked with anti-CD16/CD32 (eBioscience) for 15 min at room temperature (RT). Fluorescent-labeled antibodies targeting CD11b (eBioscience), F4/80 (eBioscience), CD64 (BioLegend), CD71 (BD Pharmingen) and CD86 (eBioscience) were incubated for 20 min at RT. Stained cells were resuspended in PBS with 0.5% bovine serum albumin and 2.5 mM EDTA and measured on a Beckman Coulter CytoFLEX and analyzed with FlowJo software V.10. Debris and doublets were excluded using forward and side scatter. CD11b+ and F4/80+ cells were considered as PMs.

### RNA sequencing

Total RNA was isolated from PMs using the RNeasy Mini Kit (QIAGEN) with DNase treatment. RNA (700 ng) was used for Illumina library construction. RNA amplification, cDNA generation, and adaptor ligation were performed using the KAPA mRNA HyperPrep Kit (Roche) following the manufacturer’s instructions. Samples were pooled, diluted to 10 nM and sequenced single end on an Illumina HiSeq 4000 instrument (Illumina) to a depth of ±20 million reads with a length of 50 base pairs.

### Bioinformatics

Reads were aligned to the mouse genome mm10 by STAR 2.5.2b with default settings.^14^ Binary alignment map (BAM) files were indexed and filtered on MAPQ>15 with SAMTools 1.3.1.^15^ Raw tag counts and reads per kilobase million (RPKM) per gene were summed using HOMER2’s analyzeRepeats.pl script with default settings and the -noadj or -rpkm options for raw counts and RPKM reporting.^16^ Differential expression was assessed using the DESeq2 Bioconductor package in an R V.3.4.3 programming environment with gene expression called differential with a false discovery rate (FDR) <0.05 and a median RPKM>1 in at least one group.^17^ Presented RPKM values were tested using one-way analysis of variance (ANOVA) followed by Bonferroni’s post hoc comparisons test. Pathway enrichment analysis was performed using Metascape.^18^ Gene Ontology biological processes were selected for the analysis. Upstream regulator analysis was performed using Ingenuity Pathway Analysis software (QIAGEN).

### Weighted gene coexpression network analysis

A weighted correlation network was created using the weighted gene coexpression network analysis (WGCNA) R software package.^19 20^ Transcripts with a median RPKM>1 in at least one group of unstimulated PMs were selected for WGCNA. For network construction, an adjacency matrix was calculated with a soft-thresholding power of 8. Signed adjacency was used for clustering and the signed hybrid model was selected as the network type. Next, to calculate the connection strength between all transcript pairs, a topological overlap measure (TOM) dissimilarity matrix (1-TOM) was used for average hierarchical clustering. Module eigengenes were calculated by summarizing the first principal component of the modules. Further, the correlation between the transcript expression profile and the module eigengene, also known as the module membership, was calculated. Next, the module-trait association was used to identify clinically relevant modules related to bodyweight (trait). For this, the correlation between the module eigengene and bodyweight was used. The gene significance (GS) measure between the expression profile and trait was calculated for every transcript and allows identification of proteins, which are strongly associated with the trait bodyweight. Hub genes, which are strongly associated with the trait bodyweight (GS>0.7) and with a module membership >0.7, were selected for the creation of a functional protein association network. The search tool for the retrieval of interacting genes/proteins (STRING) database was used for the creation of this network with high confidence (0.7).^21^

### Statistical analysis

For all experiments, five to seven mice were used per group. Outliers were identified with the robust regression and outlier detection (ROUT) method with Q=1% and subsequently removed. Normality of the data was tested using D’Agostino-Pearson and Shapiro-Wilk tests. Data were further analyzed by one-way ANOVA, two-way ANOVA, Kruskal-Wallis tests, unpaired t-test or Mann-Whitney test using GraphPad Prism V.7.03 (GraphPad Software). Tukey and Sidak multiple correction tests were applied when necessary. P<0.05 was considered significant. Data are presented as means±SD.

## Results

### Weight loss reverses classical DIO and T2D characteristics

**Figure 1:**
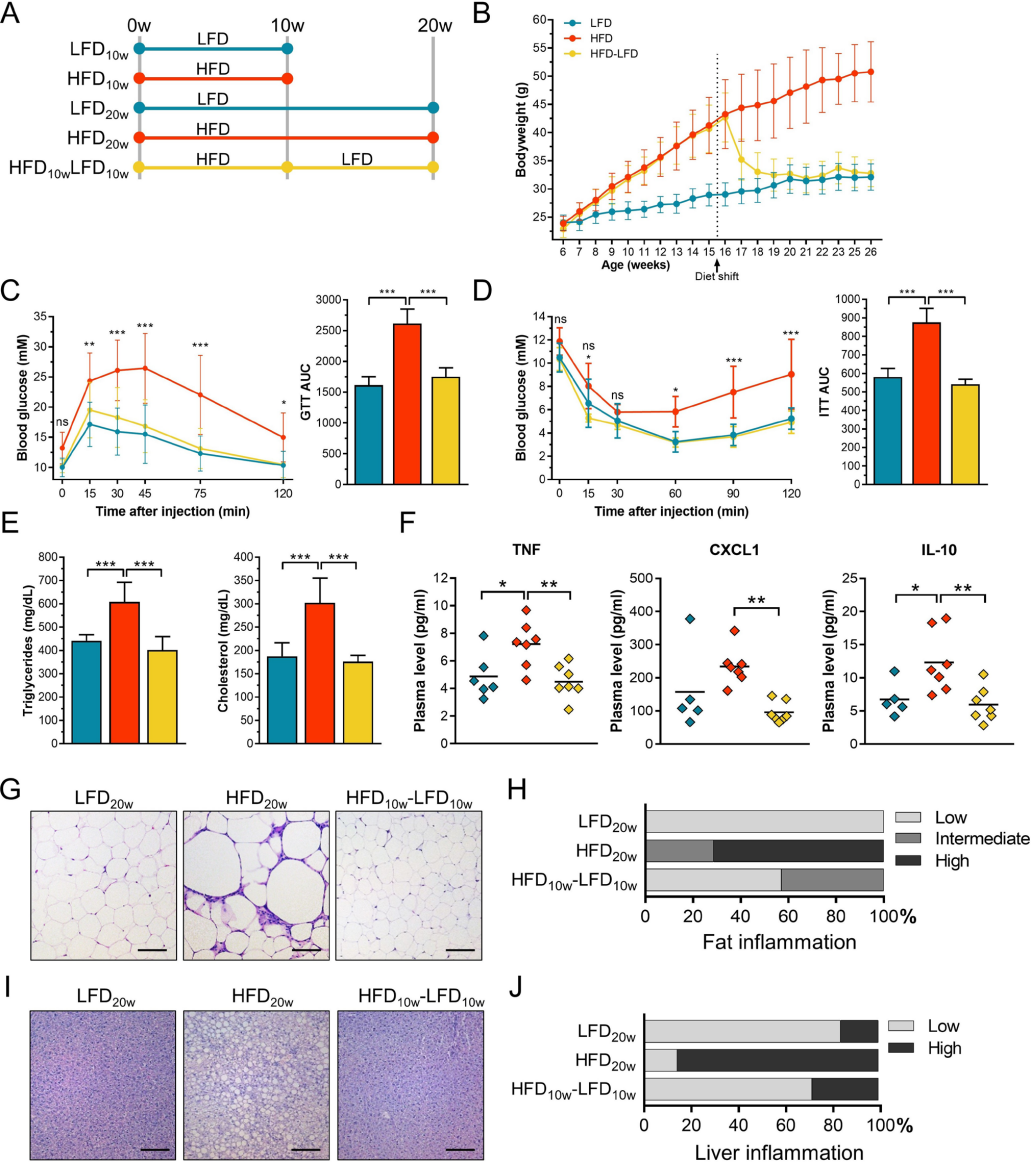
Diet-induced obesity (DIO) and type 2 diabetes (T2D) characteristics were reversed by subsequent weight loss. (A) Male C57BL/6 mice were fed a LFD (n=24) or HFD (n=42) diet for 10 weeks when 12 LFD and 14 HFD mice were sacrificed. Fourteen of the remaining HFD mice were shifted to a LFD for 10 weeks. The rest of the mice continued with their original diet. (B) Mouse bodyweight was measured once a week. (C) Blood glucose levels were measured over time after 5 hours of fasting and subsequent intraperitoneal injections of glucose. Glucose area under the curve (AUC) was calculated per group. (D) Blood glucose levels were measured over time after 4 hours of fasting and subsequent intraperitoneal injections of insulin. Glucose AUC was calculated per group. (E) Fasting plasma triglycerides and cholesterol levels. (F) Plasma cytokines (TNF, CXCL1, and IL-10) were measured using an electrochemiluminescence assay. (G) H&E stained visceral fat sections. Scale bar length: 100 µm. (H) H&E stained fat sections were scored for the count of infiltrating immune cells as a measure of inflammation. (I) H&E stained liver sections. Scale bar length: 100 µm. (J) H&E stained liver sections were scored for the count of infiltrating immune cells as a measure of inflammation. Error bars represent SD. AUC, area under the curve; CXCL1, C-X-C motif chemokine ligand 1; GTT, glucose tolerance test; HFD, high-fat diet; IL-10, interleukin-10; ITT, insulin tolerance test; LFD, low-fat diet; TNF, tumor necrosis factor.

To study HFD-induced consequences followed by weight loss, we used dietary interventions to investigate mouse PMs. Male C57BL/6 mice were fed a HFD or LFD for 10 weeks. After 10 weeks, a subset of the mice was sacrificed. Next, a part of the residual HFD mice was switched to a LFD for another 10 weeks (HFD-LFD). The remaining mice continued with their original diet (figure 1A). After 2 weeks, the HFD mice gained significantly more weight compared with LFD mice (figure 1B). From 1 week after the diet shift, there was no significant bodyweight difference remaining between HFD-LFD and LFD mice. GTT and ITT showed significantly increased blood glucose levels at several time points in fasted HFD mice after 8–9 weeks (online supplementary figure S2A,B) and 18–19 weeks of the diet (figure 1C,D). There was no difference in blood glucose levels between LFD and HFD-LFD mice at any time point. Fasting plasma triglyceride and cholesterol levels were significantly increased in HFD mice after 8 weeks (online supplementary figure S2C) and 18 weeks (figure 1E) and recovered completely after the diet shift. To summarize, HFD induces various obesity and T2D characteristics which disappear after weight loss.

In obesity, expanded adipose tissue contributes to a state of systemic low-grade inflammation.^10 11^ To confirm this chronic inflammatory state and to determine whether this state was reversible by weight loss, various plasma cytokines and chemokines were measured and immune cell quantity was analyzed in liver and visceral fat. TNF, CXCL1, and IL-10 were significantly increased after 20 weeks of HFD (figure 1F). All cytokine and chemokine levels recovered after weight loss. Adipose tissue of HFD mice contained enlarged adipocytes with an increased number of immune cells (figure 1G,H). After weight loss, adipocyte size decreased and additional immune cells disappeared. Similar effects were seen for hepatic immune cell infiltration and lipid accumulation (figure 1I,J). To conclude, weight loss repairs the HFD-induced chronic inflammatory state.

### HFD primes PMs to a deactivated state that disappears after weight loss

**Figure 2:**
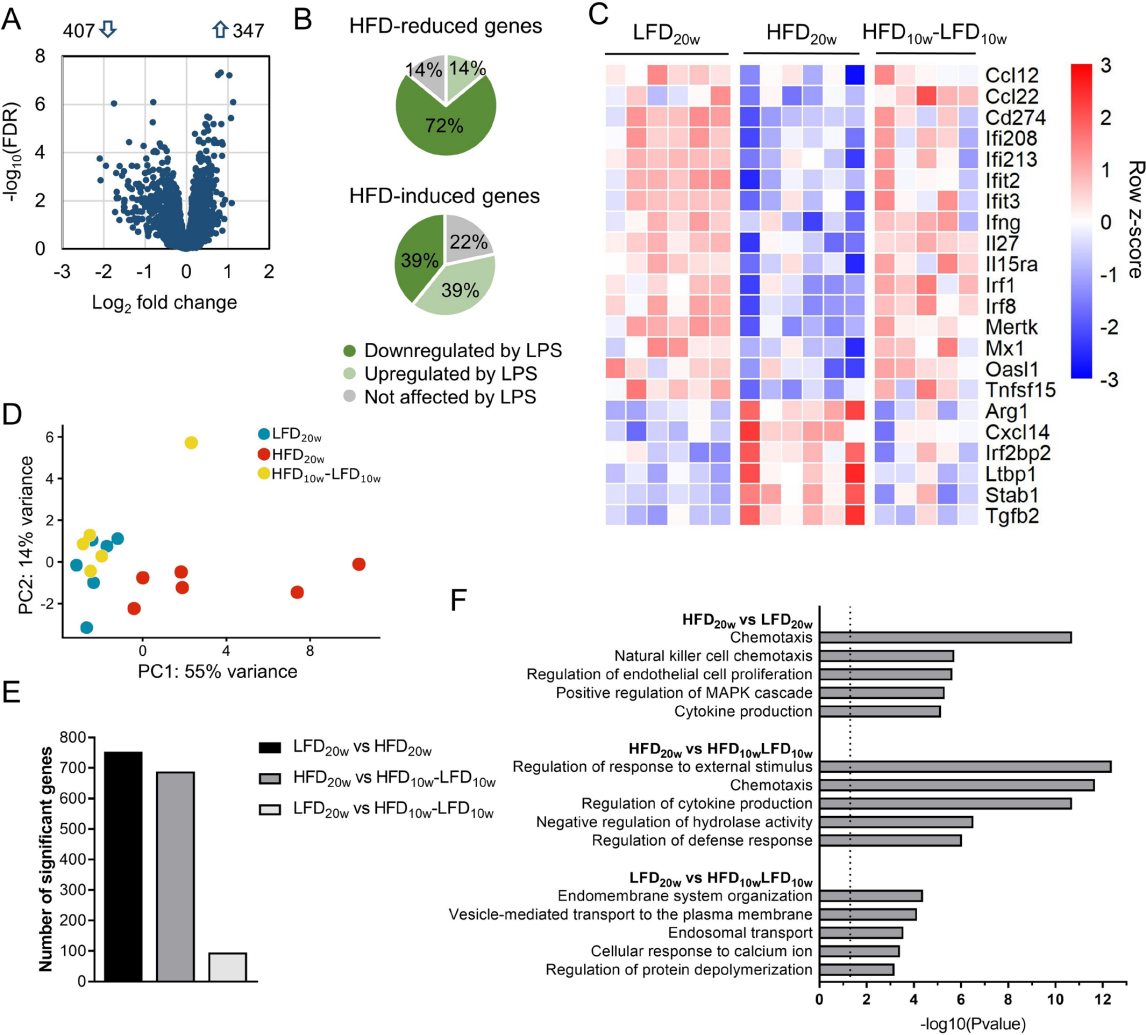
Transcriptomic profiles of peritoneal macrophages (PM) indicated an impaired immune response caused by HFD which disappeared after weight loss. (A) Volcano plot of the gene expression of LPS-stimulated PMs of HFD versus LFD mice. (B) Pie charts of HFD-reduced and HFD-induced genes (FDR<0.05) and the overlap with the normal (LFD) LPS response. (C) Heatmap with the row Z-score of significantly differentially (FDR<0.05) regulated immune response genes of RNA-seq data of LPS-stimulated PMs. (D) Principal component analysis (PCA) of each biological RNA-seq replicate of LFD, HFD, and HFD-LFD. (E) The number of significantly differentially regulated genes per comparison (FDR<0.05). (F) Top five pathways of Gene Ontology pathway enrichment analysis of the top 95 differentially expressed genes of each comparison from (E). FDR, false discovery rate; HFD, high-fat diet; LFD, low-fat diet; LPS, lipopolysaccharide.

To determine whether the macrophage response against pathogens was affected by obesity, we examined the transcriptional profiles of PMs, either under unstimulated conditions or on LPS-induced activation. As a baseline, the LPS response of PMs from LFD mice was analyzed using RNA-seq. After administration of LPS, 4654 genes were significantly upregulated and 4636 genes were significantly downregulated (FDR<0.05; online supplementary figure S3A). As expected, transcriptional levels of important immune regulators such as *Il1b*, NO synthase 2 (*Nos2*), *Il6*, and *Ifng* were significantly induced after LPS exposure. Next, the transcriptomes of LPS-stimulated PMs of LFD mice were compared with those of HFD mice to assess the impact of obesity on bacterial infection. We found 347 significantly upregulated genes and 407 significantly downregulated genes in HFD PMs compared with LFD PMs (FDR<0.05; figure 2A). Subsequently, we analyzed which part of the HFD-affected genes are at play in the control response to LPS under LFD conditions and whether these differentially regulated transcripts are enhanced or dampened in the LPS response. For instance, genes that are significantly upregulated by LPS administration in LFD mice may be significantly less induced in HFD mice which indicates a dampened LPS response. Interestingly, we found that 72% of the genes reduced by HFD dampened the LPS response, while 14% were not affected by LPS administration (figure 2B). Besides, 39% of the genes induced by HFD also dampened the LPS response, while 22% were not changed by LPS exposure. Our data thus suggest a predominant suppression of the LPS response of PMs in DIO and T2D. Genes included in this suppression encompassed highly relevant proinflammatory regulators like interferon regulatory factor 8 (*Irf8*), *Ifng* and chemokine (C-C motif) ligand 12 (*Ccl12*) (figure 2C). Moreover, HFD induced upregulation of classical anti-inflammatory mediators such as arginase 1 (*Arg1*) and transforming growth factor beta 2 (*Tgfb2*). The LPS-induced gene expression of the genes depicted in figure 2C is shown in the online supplementary figure S3B. Interestingly, the effects on these and other highly relevant inflammation controlling genes completely reversed after the diet shift (figure 2C). Focusing on the reversibility of HFD-affected gene expression, we found that the principal component analysis reveals two separated clusters: one cluster that consists of overlapping LFD and HFD-LFD samples, which is moderately separated from a cluster which contains samples from the HFD PMs (figure 2D), indicating reversibility on diet switch. Moreover, there were only 95 significantly differentially regulated genes between the LFD versus HFD-LFD PMs after LPS exposure, while 689 and 754 genes were differentially regulated between HFD versus HFD-LFD and HFD versus LFD PMs (figure 2E). Pathway enrichment analysis on the top 95 differentially regulated genes of each comparison clearly showed that the immune response of PMs is affected by HFD (figure 2F). This enrichment immune-related pathway did not exist in LFD versus HFD-LFD PMs and thus again showing a reversible profile. Under unstimulated conditions, samples clustered similarly (online supplementary figure S3C). Seven hundred and fifty-five unique genes were significantly differentially expressed between HFD and LFD without LPS exposure (FDR<0.05). Six hundred and eighty-three genes were found to be significantly differentially expressed between HFD and HFD-LFD. Pathway enrichment analysis revealed that also under basal conditions, the inflammatory response of PMs was affected by HFD (online supplementary figure S3D). Taken together, our data show that DIO and T2D restrict LPS-induced immune activation of PMs. Interestingly, this effect is already observed prior to LPS stimulation, so it seems that a HFD primes PMs towards a deactivated phenotype which is translated to an impaired LPS response.

**Figure 3:**
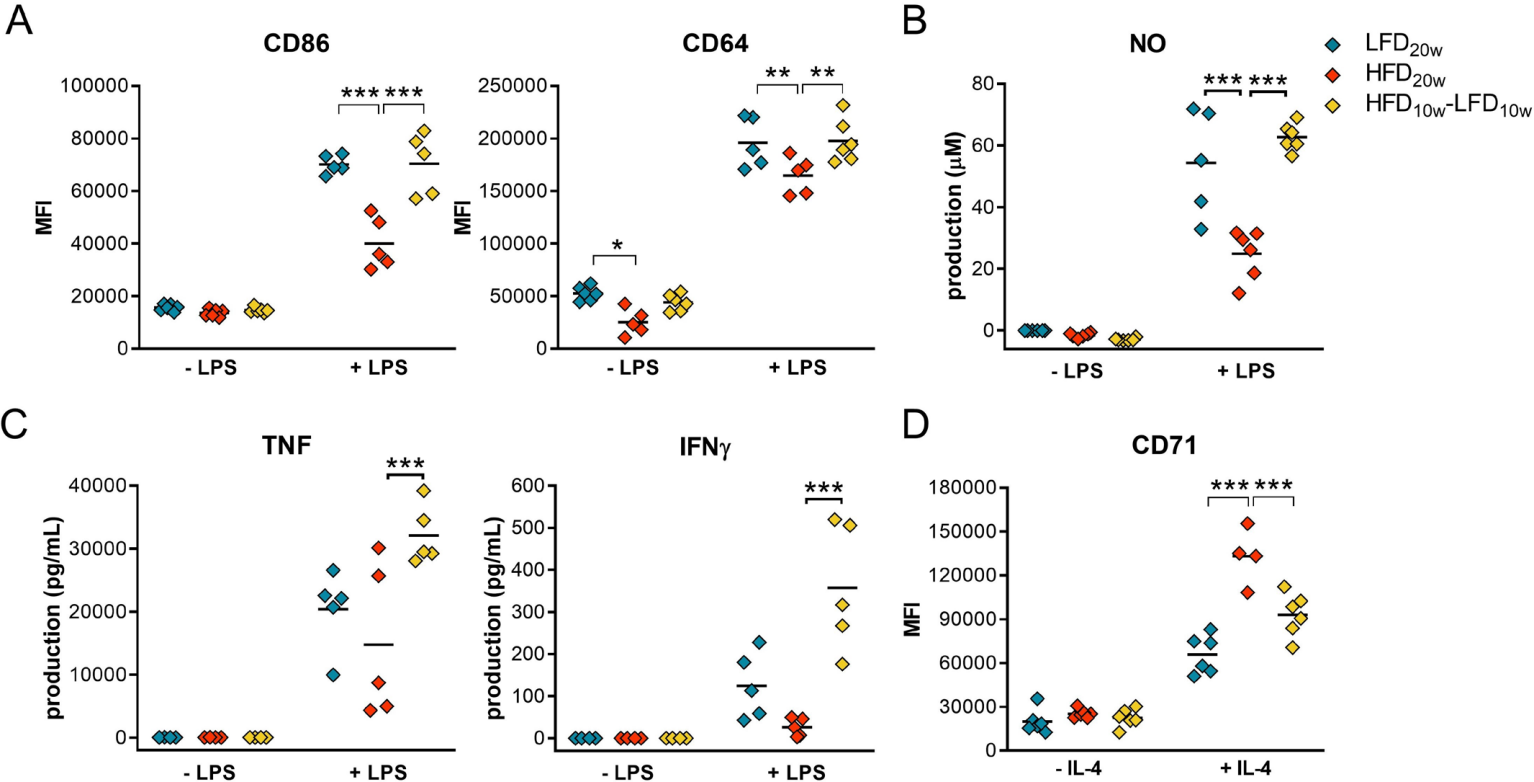
HFD peritoneal macrophages (PM) were primed to a less proinflammatory state and this state was reversed by weight loss. (A) Surface expression of CD86 and CD64 on PMs stimulated with and without 24 hours of LPS was determined using flow cytometry. (B) PM NO secretion was measured using a Griess reaction. (C) TNF and IFNγ production by PMs was quantified by an electrochemiluminescence assay. (D) Surface expression of CD71 on PMs stimulated with and without IL-4 for 24 hours was measured by flow cytometry. HFD, high-fat diet; IFNγ, interferon gamma; IL-4, interleukin-4; LFD, low-fat diet; LPS, lipopolysaccharide; NO, nitric oxide; TNF, tumor necrosis factor.

To confirm this deactivated immune response, proinflammatory surface markers and cytokine secretion were measured of LPS-stimulated PMs. We found a significantly lower surface expression of costimulatory molecule CD86 and Fc receptor CD64 on HFD compared with LFD and HFD-LFD PMs (figure 3A), also after 10 weeks of HFD (online supplementary figure S4A). Moreover, CD64 expression was also significantly lower without LPS stimulation. HFD PMs secreted less TNF, IFNγ, and NO, which was reversed after weight loss (figure 3B,C). Besides, NO secretion was also significantly decreased after 10 weeks of HFD (online supplementary figure S4B). Simultaneously, we found a significantly increased expression of the anti-inflammatory surface receptor transferrin (CD71) on HFD PMs after IL-4 stimulation (figure 3D). These results confirm that HFD primes PMs towards a less proinflammatory state and that this state is reversible by a diet shift.

### Weight loss reverses the obesity and T2D affected metabolism of PMs

**Figure 4:**
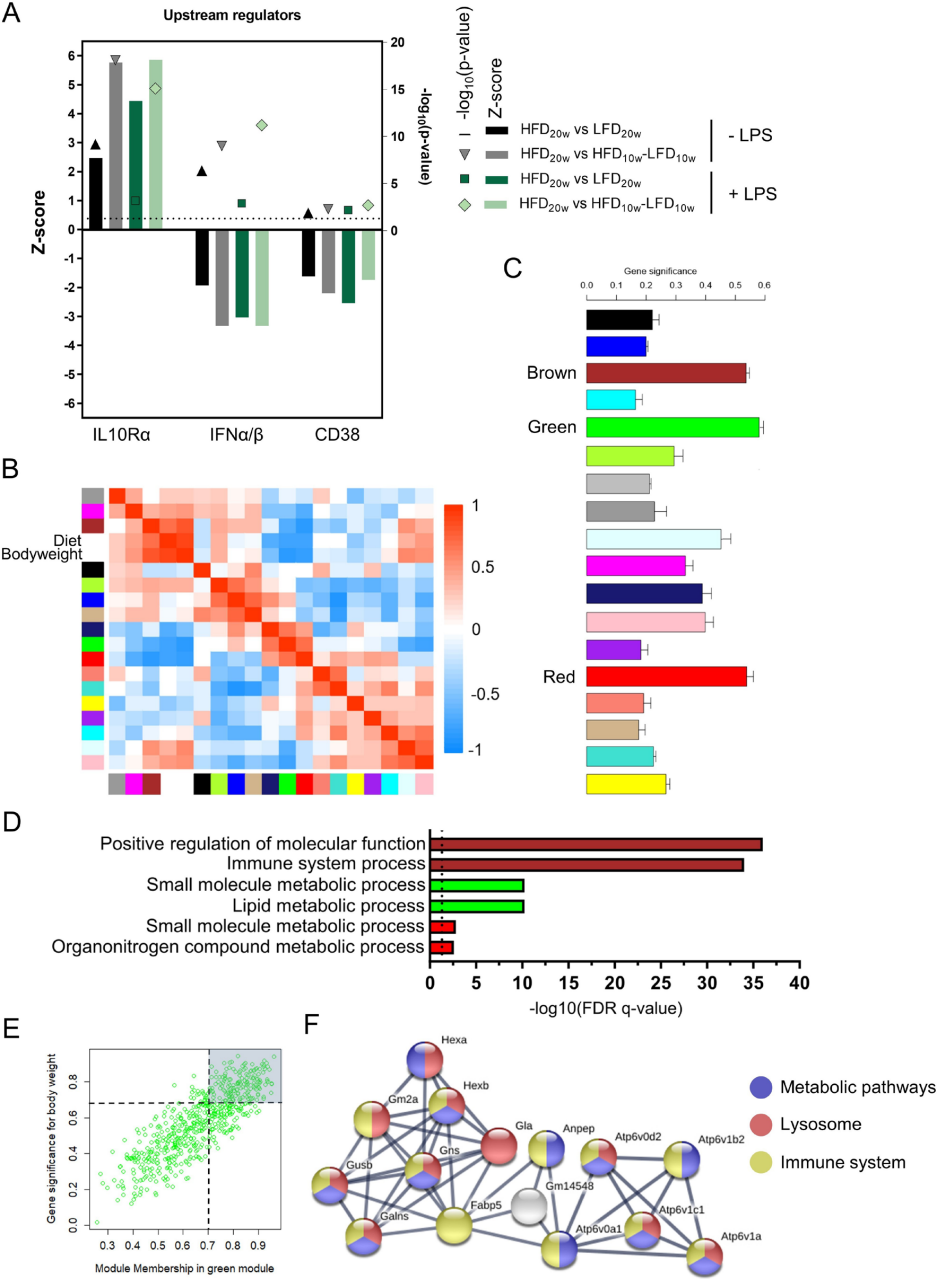
Peritoneal macrophage (PM) metabolism is affected by diet-induced obesity (DIO) and diabetes and is reversible by a shift to LFD. (A) Upstream regulator analysis of Ingenuity Pathway Analysis (IPA) on differentially regulated genes (FDR<0.1). (B) Module eigengene adjacency heatmap. Module eigengenes were calculated by summarizing the first principal component of the modules. (C) Mean gene significance (GS) across all genes in a module. A large absolute value of the gene significance measure corresponds to a small two-sided P value. (D) Top five pathways of Gene Ontology pathway enrichment analysis of the brown, green, and red modules. (E) Scatter plot of the module membership in the green module against the GS for bodyweight. (F) Largest functional protein association network of the green module with high confidence (0.7). Hub genes with a module membership in the green module and an absolute GS for bodyweight >0.7 were selected for this network (162 genes). FDR, false discovery rate; HFD, high-fat diet; IFN, interferon; IL10, interleukin-10; LFD, low-fat diet; LPS, lipopolysaccharide.

To gain more knowledge about the cause and mechanism of the deactivated PM phenotype, we applied computational strategies. First, upstream regulator prediction analysis was performed on the significantly differentially regulated genes of HFD versus LFD and HFD versus HFD-LFD PMs with and without LPS. Activation by anti-inflammatory upstream regulator IL10Rα and suppression of proinflammatory upstream regulators IFNα/β and CD38 were predicted to play a significant role in the anti-inflammatory phenotype of the cells (figure 4A).^22^

Next, we constructed a coexpression network with the transcriptional data of the three different groups of unstimulated PMs. WGCNA identified 18 modules of similar expression obtained by hierarchical clustering with adjacency-based dissimilarity (online supplementary figure S5A). The brown module appeared to have the strongest positive correlation with the incorporated experimental traits diet and bodyweight whereas the green and red modules showed the strongest negative correlation (figure 4B). Genes present in those three modules appeared to have the highest mean GS (figure 4C). Pathway enrichment analysis indicated that genes present in the brown module play a role in the immune system (figure 4D). Remarkably, genes of the green module showed enrichment for metabolic processes like lipid metabolism which was not demonstrated with pathway enrichment analysis on all significantly differentially regulated genes (figure 2F). Transcripts in the red module were, however, less significantly, also assigned to metabolic pathways. A heatmap of the significantly differentially regulated genes of these two metabolic pathways confirmed the HFD-induced metabolic changes (online supplementary figure S5B). Next, we found the GS for bodyweight in the green module to be highly correlated (R=0.83) with the module membership (similarity of a gene to the module eigengene; Figure 4E). This demonstrates that genes significantly associating with bodyweight are also the most prominent transcripts of the green module. We found that protein-coding transcripts with a high GS and module membership of the green module form a significant protein-interaction network (figure 4F). This network encloses several V-type ATPases (ATP6V) and other proteins with metabolic, lysosomal and immune-related functions. Taken together, HFD affects PM metabolism which may play a role in the deactivated phenotype of PMs in obesity. We also found that this metabolic interruption directly correlates with bodyweight and disappears after a shift to LFD.

Spann *et al* described that a deactivated macrophage phenotype could be attributed to deregulated cholesterol synthesis pathways.^23^ Therefore, we next focused our analysis on these processes. Indeed, we found a significant downregulation of enzymes of the cholesterol biosynthesis, such as 3-hydroxy-3-methylglutaryl-CoA synthase 1 (*Hmgcs1*), lanosterol synthase (*Lss*), and 24-dehydrocholesterol reductase (*Dhcr24*) expression (online supplementary figure S5C). Dhcr24 catalyzes the reduction from desmosterol to cholesterol and its downregulation has been shown to lead to accumulation of desmosterol consequently activating liver X receptors (LXR) and sterol regulatory element-binding proteins (SREBP) target genes and suppressing inflammatory responses. In agreement, we found an upregulation of numerous LXR and SREBP targets in the HFD PMs, such as *Idol*, *Srebf1*, *Srebf2*, *Scd1*, and *Abca1* (online supplementary figure S5C), as well as a downregulation of numerous proinflammatory genes (figure 2C). Furthermore, we found no clear activation or suppression of glycolysis and the pentose phosphate pathway (PPP).

## Discussion

Obesity and T2D affect the immune system and thereby play an important role in the pathophysiology of obesity-related comorbidities like infection susceptibility. In this study, we focused on the effects of obesity and T2D on PMs since macrophages play a key role in defense against invading pathogens.^24^ For example, it has been shown that infection susceptibility to *Mycobacterium tuberculosis* and *Streptococci* spp correlates with a decreased NO and TNF production by macrophages.^25 26^ We also determined whether the HFD-induced effects were reversible after weight loss because it is not clear which obesity-related complications will resolve after weight loss and which need additional treatment. Contrary to the overbalance of proinflammatory macrophages in adipose tissue in obesity and T2D, we demonstrate a deactivated state of macrophages outside the adipose tissue. Furthermore, we showed that this abnormally deactivated state of macrophages is lost after weight loss. Whether these deactivated macrophages are also present in patients with obesity and T2D and significantly contribute to their increased infection susceptibility remains to be determined. Our data suggest that weight loss in patients with obesity and T2D will restore the normal proinflammatory state of macrophages and thereby reduce immune-related comorbidities of those patients.

First, the presence and reversibility of obesity and T2D were confirmed in the different groups. As expected, mice showed obesity and T2D characteristics (eg, weight gain, insulin resistance and high levels of plasma cholesterol and triglycerides) after 10 and 20 weeks of HFD. Besides, systemic low-grade inflammation, a classical hallmark and confounding factor for comorbidities, was confirmed in HFD mice by detection of elevated plasma TNF, CXCL1 and IL-10 levels, more infiltrating immune cells together with hepatic lipid accumulation, and enlarged adipocytes. van der Heijden *et al* also found significantly higher plasma levels of TNF, CXCL1, and IL-10 in obese mice.^27^ In our studies, all changes disappeared after weight loss and our results support that the HFD induces a chronic inflammatory state which is reversible by dietary changes.

Transcriptional profiling indicated that DIO and T2D affected PM activation. We found a downregulation of proinflammatory genes and upregulation of anti-inflammatory genes in HFD PMs. Significantly lower surface expression levels of CD86 and CD64 on HFD PMs and less secretion of TNF, IFNγ and NO confirmed the deactivated PM state. Simultaneously, HFD PMs expressed significantly increased levels of CD71 after IL-4 incubation. All these differences disappeared after the diet shift. Thus, our data clearly show that a HFD dampens the LPS response of PMs and this effect disappears after weight loss. Interestingly, these changes are also observed before LPS stimulation, so it seems that PMs are tolerized by the HFD. In line with our findings, Amar *et al* showed that *Porphyromonas gingivalis* exposed PMs from mice with DIO produced less TNF, IL-1β, and IL-6.^28^ Similarly, Ieronymaki *et al* demonstrated that alveolar macrophages from insulin-resistant mice produced significantly less TNF after LPS exposure and expressed more arginase 1.^29^ They also showed a reduced inflammatory response after a cecal ligation and puncture (polymicrobial sepsis model) in diabetic mice.

Several computational strategies were applied to unravel the cause of the deactivated PM phenotype. Upstream regulator prediction analysis predicted an upregulation of upstream regulator IL10Rα and a downregulation of IFNα/β and CD38 coinciding with the less inflammatory phenotype of HFD PMs. Indeed, we and others found higher plasma IL-10 levels in obese mice, which can trigger the IL-10 receptor and induce a deactivated macrophage phenotype.^30^ Second, type 1 IFNs protect the host from viral infections and induce myeloid-mediated bacterial clearance.^31 32^ Furthermore, Matalonga *et al* found that the number of infected macrophages and intracellular bacteria was increased in CD38-deficient macrophages, which coincides with the predicted downregulation of CD38 and a flawed LPS response in HFD PMs.^33^ Notwithstanding, other factors, or a combination of factors, like the richly present unsaturated fatty acid oleic acid of the HFD may also contribute to the deactivated macrophage phenotype.^34 35^

WGCNA identified HFD-induced metabolic alterations to correlate with bodyweight. These metabolic changes may play a role in the mechanism of the deactivated phenotype since metabolism and macrophage function are highly dependent.^36^ For instance, it has been shown that T2D promotes an impaired control of *M. tuberculosis* in human foamy macrophages by inducing lysosomal dysfunction.^37^ Additionally, previous work has shown that PMs from Ldlr^−/−^ mice fed a Western diet show a deactivated phenotype.^23 38^ These macrophages differentiate in a cholesterol-rich and atherosclerotic environment and will become lipid-laden foam cells. Their deactivated phenotype is explained by the accumulation of desmosterol^23^ and a disrupted PPP.^38^ In our current studies, we have focused on a DIO model with insulin resistance and in this model, it has been shown that PMs do not acquire foam cell characteristics.^39^ Similarly, as we demonstrated in our mice, patients with obesity and T2D may also encounter immune-related complications in the absence of atherosclerosis and hypercholesterolemia. Despite the absence of foam cells, we hypothesize that LXR-dependent and SREBP-dependent mechanisms might explain our deactivated PM phenotype. In accordance with Spann *et al*, we found a significant downregulation of several enzymes of the cholesterol biosynthesis pathway, including *Dhcr24*.^23^ Downregulation of Dhcr24 has been shown to result in accumulation of desmosterol which can activate LXR and SREBP target genes. Furthermore, it has been shown that LXR activation inhibits inflammatory responses. In agreement with Spann *et al*, we found an upregulation of numerous LXR and SREBP targets in the HFD PMs, as a downregulation in numerous proinflammatory genes. Thus, LXR-dependent and SREBP-dependent mechanisms may explain our deactivated PM phenotype.

Inflammation plays a role in host defense against pathogens and impacts the development and progression of cancer. Michelet *et al* recently found that obesity also limits the antitumor response of natural killer cells in mouse and human.^40^ Furthermore, tumor-associated macrophages (TAM) have anti-inflammatory characteristics that promote tumor immune evasion and maintain tumors by specific cytokine secretion that increases tumor cell viability and facilitates metastasis.^41^ On the other hand, proinflammatory macrophages have been shown to inhibit tumor growth and induce cell death. For instance, the CD86–CD28 interaction has been shown to play an important role in the induction of T-cell-mediated tumor killing. Moreover, low numbers of CD86^+^ TAMs have been correlated with an aggressive tumor phenotype in patients.^42^ Since we identified anti-inflammatory macrophages in obesity, with significantly lower CD86 expression, and anti-inflammatory macrophages are known to significantly contribute to cancer progression, we speculate that these macrophages may affect cancer progression in patients and that weight loss will be beneficial in this respect as well.

Hyperglycemic memory is a term that has been used to describe the occurrence that despite maintaining normal blood glucose levels after a period of hyperglycemia, a subset of the patients with diabetes develops comorbidities nonetheless.^43–45^ This hyperglycemic memory phenomenon can be a result of epigenetic changes caused by hyperglycemia which persist after normoglycemia.^46^ For example, Rodrigues *et al* showed with db/db mice that T2D can result in stem cell aberrations which directly affect tissue function and persist after return to normoglycemia.^9 47^ Still, Siersbæk *et al*, who used a model more similar to ours, found that the HFD-induced changes of mouse hepatic transcription and enhancer activity were reversible after weight loss, which is more consistent with our findings.^48^ Future research should focus on hyperglycemic memory and determine when this phenomenon is existing and when not. This will be very useful for the treatment of obese individuals with T2D and follow-up care after the maintenance of normal blood glucose levels.

In summary, contrary to the proinflammatory phenotype of macrophages in adipose tissue in obesity and T2D, we demonstrate a deactivated state of macrophages outside the adipose tissue. Suppressed macrophage activity may contribute to the impaired immune response against pathogens and increased prevalence of infections that are seen in obese and T2D individuals. Finally, we show beneficial effects of weight loss in these settings by reversal of the deactivated macrophage phenotype.
