# Supplementary material for: Peritoneal macrophages have an impaired immune response in obesity which can be reversed by subsequent weight loss
Source: BMJ Open Diabetes Res Care. 2019 Nov 2;7(1):e000751. doi: 10.1136/bmjdrc-2019-000751 (PMC6861071; doi:10.1136/bmjdrc-2019-000751)

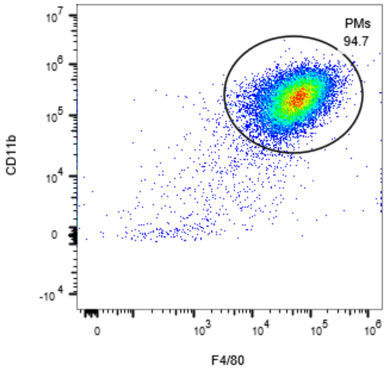

Figure S1. Flow cytometry analysis indicated a clean PM population
Flow cytometry staining for CD11b and F4/80 surface makers. A representative image is shown.

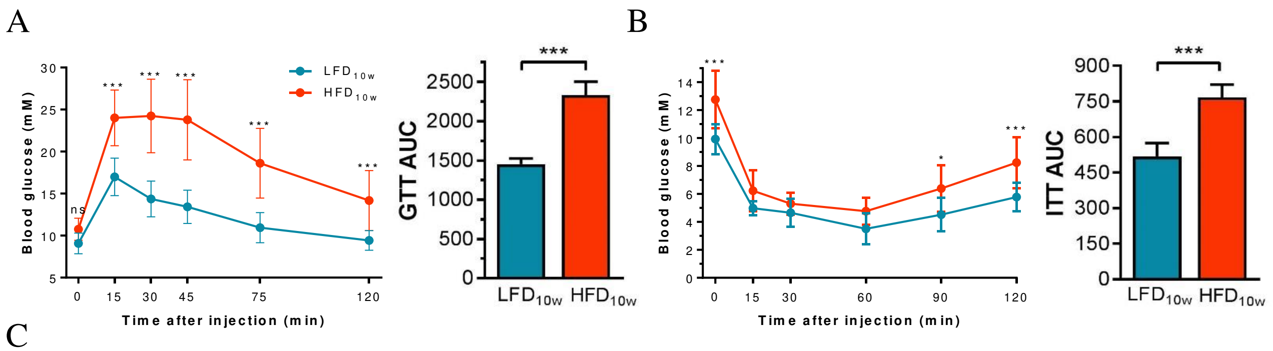

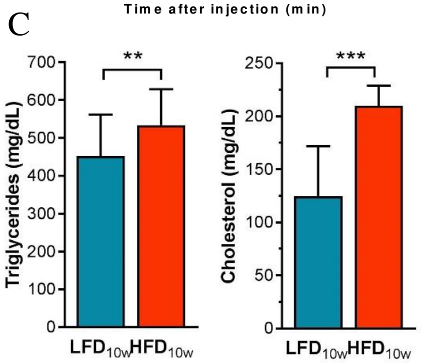

Figure S2. DIO and T2D characteristics were confirmed in HFD mice

(A) GTT after 8 weeks of the diet. Blood glucose levels were measured over time after 5 hours of fasting and subsequent i.p. injection of glucose. Glucose area under the curve (AUC) was calculated per group.

(B) ITT after 9 weeks of the diet. Blood glucose levels were measured over time after 4 hours of fasting and subsequent i.p. injection of insulin. Glucose AUC was calculated per group.

(C) Fasting plasma triglycerides and cholesterol levels after 8 weeks of the diet.

Error bars represent standard deviation (SD).

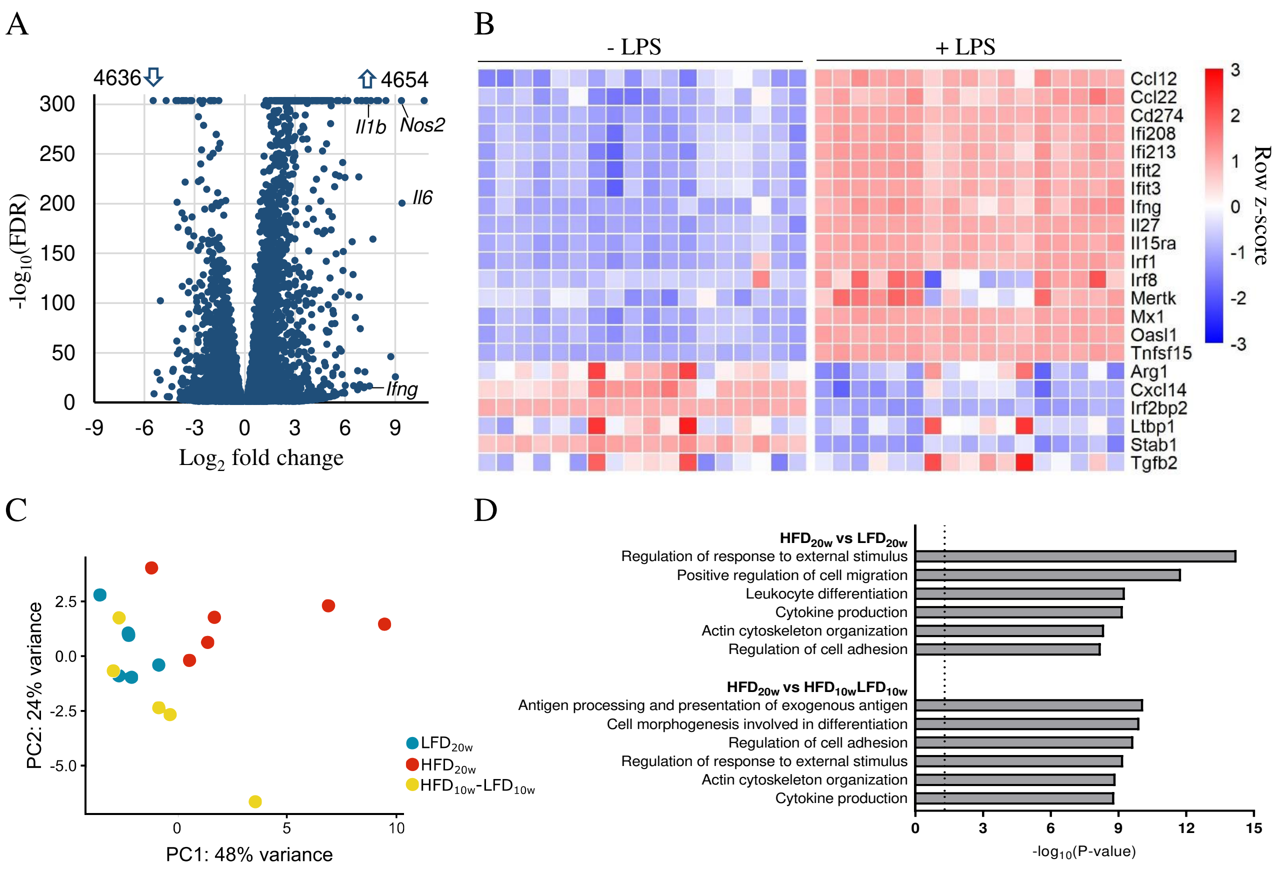

Figure S3. Transcriptomic profiles of PMs indicated an impaired immune response caused by HFD which disappeared after weight loss

(A) Volcano plot of the gene expression of unstimulated vs LPS-stimulated PMs of LFD mice.

(B) Heatmap with the row Z-score of immune response genes of RNA-seq data of PMs with and without 3h of LPS stimulation.

(C) PCA of each unstimulated biological RNA-seq replicate of LFD, HFD, and HFD-LFD.

(D) Top 6 pathways of Gene Ontology pathway enrichment analysis of differentially (FDR<0.05) expressed genes of each comparison of unstimulated PMs.

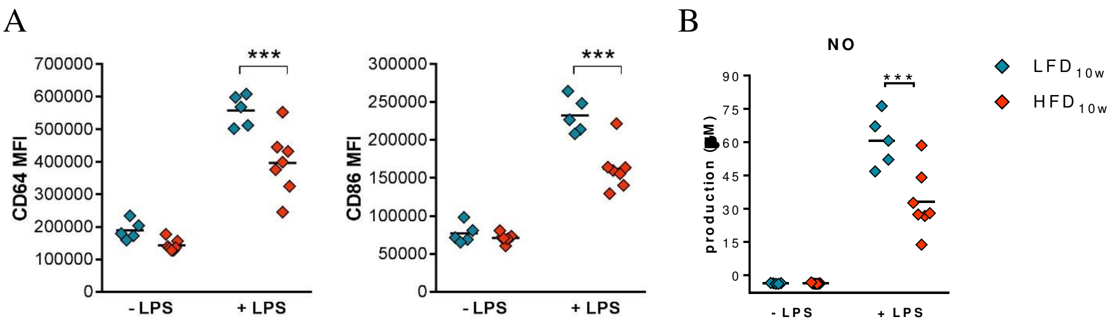

Figure S4. HFD PMs were primed to a less pro-inflammatory state
(A) Surface expression of CD64 and CD86 on PMs stimulated with and without 24 hours of LPS was determined using flow cytometry after 10 weeks of the diet.
(B) PM NO-secretion was measured using a Griess reaction after 10 weeks of the diet.

A

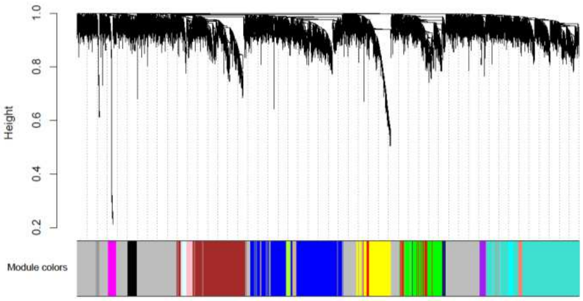

B

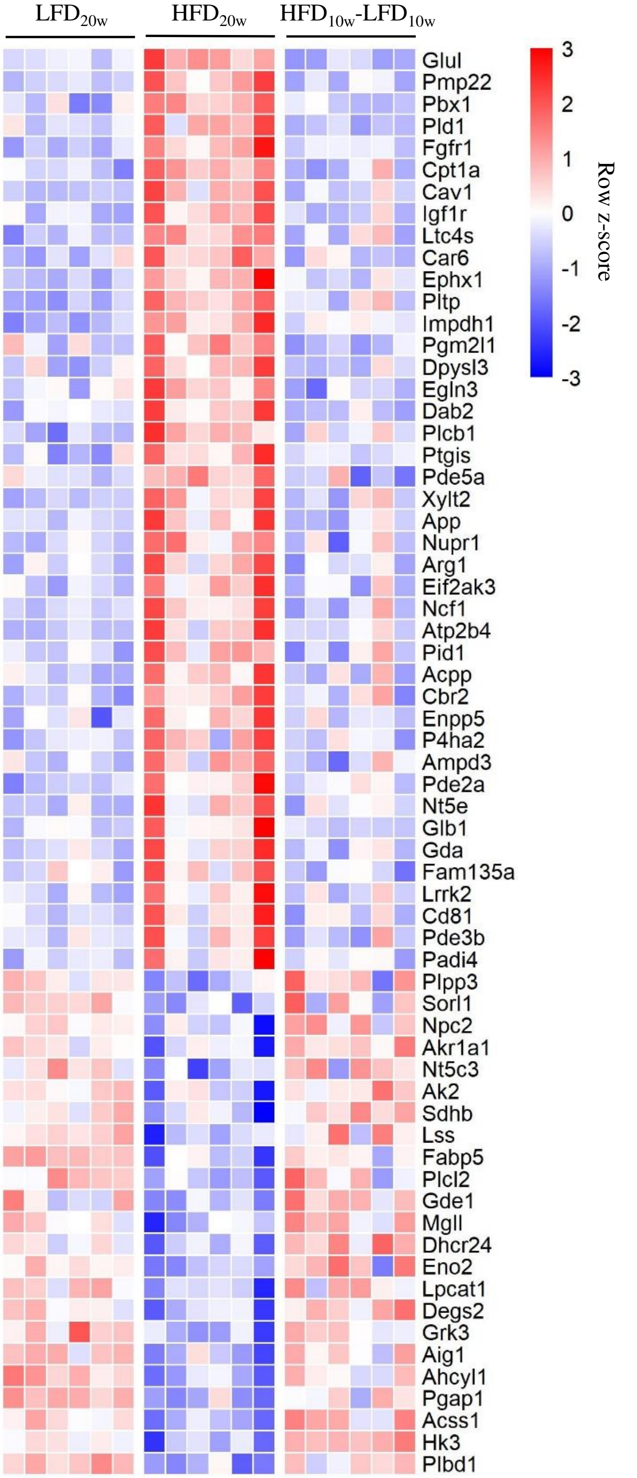

Figure S5. HFD-induced metabolic and immune regulatory dysfunctions were identified by WGCNA

(A) Gene dendrogram obtained by hierarchical clustering of adjacency-based dissimilarity of unstimulated PMs.

(B) Heatmap of genes of the pathways: small molecule metabolic process and lipid metabolic process of unstimulated PMs (FDR<0.1)

(C) Heatmap of the cholesterol biosynthesis, LXR, and glycolysis and PPP gene expression (FDR<0.1).

C

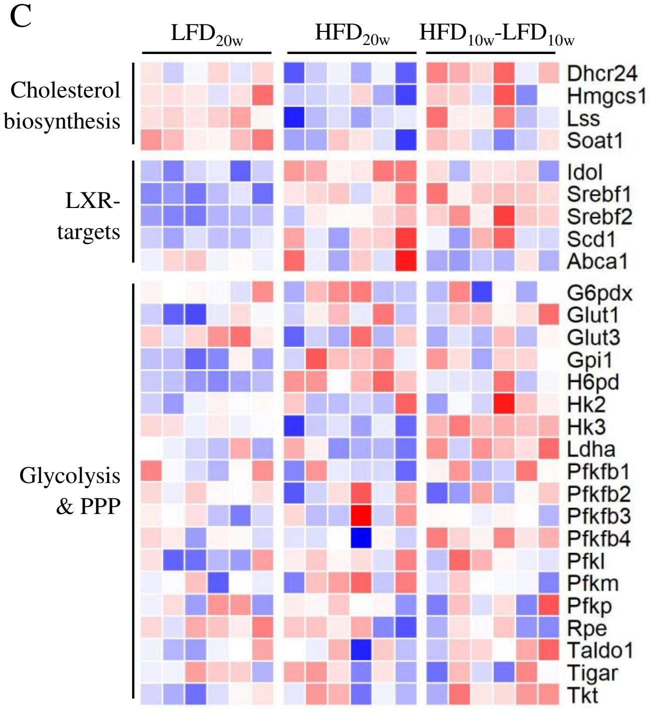

Supplement: Supplementary data [file bmjdrc-2019-000751supp001.pdf]
